# No implant migration and good subjective outcome of a novel customized femoral resurfacing metal implant for focal chondral lesions

**DOI:** 10.1007/s00167-017-4805-2

**Published:** 2017-11-22

**Authors:** Anders Stålman, Olof Sköldenberg, Nicolas Martinez-Carranza, David Roberts, Magnus Högström, Leif Ryd

**Affiliations:** 1 Department of Orthopaedics, Karolinska University Hospital, Stockholm, Sweden; 2 Department of Orthopaedics, Skane University Hospital, Malmö, Sweden; 3 Sports Medicine Umeå AB and Orthopedics, Department of Surgical and Perioperative Sciences, Umeå University, Umeå, Sweden; 4 LIME, Karolinska Institutet, Solna, Sweden; 5 Department of Orthopaedics, Danderyd Hospital, Stockholm, Sweden; 6 Stockholm Sports Trauma Research Center, Karolinska Institutet, Capio Artro Clinic, Sophiahemmet, Valhallavägen 91, 114 86 Stockholm, Sweden

**Keywords:** Focal cartilage injuries, Prosthetic inlay resurfacing, Osteochondral injury

## Abstract

**Purpose:**

Managing focal cartilage injuries in the middle-aged patient poses a challenge. Focal prosthetic inlay resurfacing has been proposed to be a bridge between biologics and conventional joint arthroplasty. Patient selection and accurate implant positioning is crucial to avoid increased contact pressure to the opposite cartilage surface. A customized femoral condyle implant for focal cartilage injuries was designed to precisely fit each patient’s individual size and location of damage. The primary objective was to assess implant safety profile, surgical usability of the implant and instruments, and implant migration with radiostereometric analysis (RSA).

**Methods:**

Ten patients 36–56 years with focal chondral defects, ICRS 3–4 of the femoral cartilage and failed earlier conservative or surgical interventions with VAS pain > 40. The patients were followed for 2 years with subjective outcome measures (VAS, EQ5D, KOOS) and RSA. The customized implant and guide instruments were manufactured by computer-aided design/computer-aided manufacturing (CAD/CAM) techniques using MRI data.

**Results:**

VAS, EQ5D and KOOS showed improvements that reached significance for VAS (*p* ≤ 0.001), Tegner (*p* = 0.034) and the KOOS subscores ADL (*p* = 0.0048), sport and recreation (*p* = 0.034) and quality of life (*p* = 0.037). VAS and KOOS scores improved gradually at 3, 6 and 12 months. The improvements in EQ5D, KOOS pain and KOOS symptoms did not reach statistical significance. No infections, deep venous thrombosis or other complications occured in the postoperative period. No radiographic signs of damage to the opposing tibial cartilage was noted. The surgical usability of implants and instruments were good. RSA did not show any implant migration.

**Conclusion:**

This is the first clinical report of a new customized, focal knee resurfacing system. The short-term implant safety and patient-related outcome measures showed good-to-excellent results.

**Level of evidence:**

Prospective case series, Level 4.

## Introduction

Focal cartilage injuries occur frequently in the knee joint and do not heal spontaneously [7, 25, 29]. Patients with focal cartilage defects often have pain and functional impairment that significantly affects quality of life [10]. These lesions might progress to osteoarthritis [6, 18, 26]. Numerous biological treatments, such as microfracture, autologous chondrocyte implantation and mosaicplasty, have been described. Short-term outcome is good especially in the young patient, but the repair tissue might degenerate over time and deteriorating results are seen with increasing patient age. These procedures are therefore not generally recommended for the middle-aged and older patients and treatment options in this group of patients have been insufficient [11, 13–15]. There is a great risk of the need of reintervention after biological treatment [12]. The management of patients who present failed attempts at biological treatment are not well reported in the literature [5]. Unicompartmental or total knee arthroplasties have a high risk of early failure in the younger patient [23].

Managing focal cartilage injuries in the middle-aged patient therefore poses a challenge [19]. Focal prosthetic inlay resurfacing has been proposed to be a bridge between biologics and conventional joint arthroplasty and could be a salvage procedure where biological treatment has failed or is considered less effective [3]. Good short time results in patient-related outcome measures are described but a high rate of revision to knee arthroplasty have been reported [8, 17]. Patient selection and accurate implant positioning is crucial to avoid increased contact pressure to the opposite cartilage surface [2, 20].

This new femoral condyle implant for focal chondral injuries together with the guide system is customized and designed to precisely be able to fit each patient’s individual size and location of the damage. The double-coated, titanium-hydroxyapatite, Co–Cr implant showed firm and consistent osseochondrointegration in an animal model [21]. The development of a customized prosthesis and guide system made accurate implant positioning possible in order to avoid damage to the opposing cartilage surface [22]. This interventional prospective consecutive cohort study describes the experiences with the first ten patients. The primary objective was to assess implant safety profile, surgical usability of implant and implant migration. The surgical technique is described, short-term outcome evaluated and implant migration is assessed with radiosterometric analysis.

## Materials and methods

Inclusion criteria were patients 30–65 years with focal chondral defects of the femoral cartilage, International Cartilage Research Society (ICRS) grade 3 or 4 on medial or lateral femoral condyles. The cartilage lesion area was ≤ 3.2 cm^2^ (diameter ≤ 2 cm). The patients had previously failed conservative or surgical interventions such as abrasions, drilling or microfracture and VAS pain > 40 with activity for more than 6 months. The patients should be capable of completing self-administered questionnaires and willing to comply with the follow-up requirements of the study. Exclusion criteria were BMI > 35 kg/m^2^, not addressed instability or other concomitant knee injuries such as meniscus injuries, apart from small flap lesions with intact rim and intact meniscal anterior and posterior horn insertions. Further exclusion criteria were established osteoarthritis, malalignment, metabolic disorders which may impair bone formation, smokers, metal allergies, inflammatory joint diseases, administration of corticosteroids, antineoplastics, immune stimulating or immunosuppressive agents.

### Implant

**Fig. 1:**
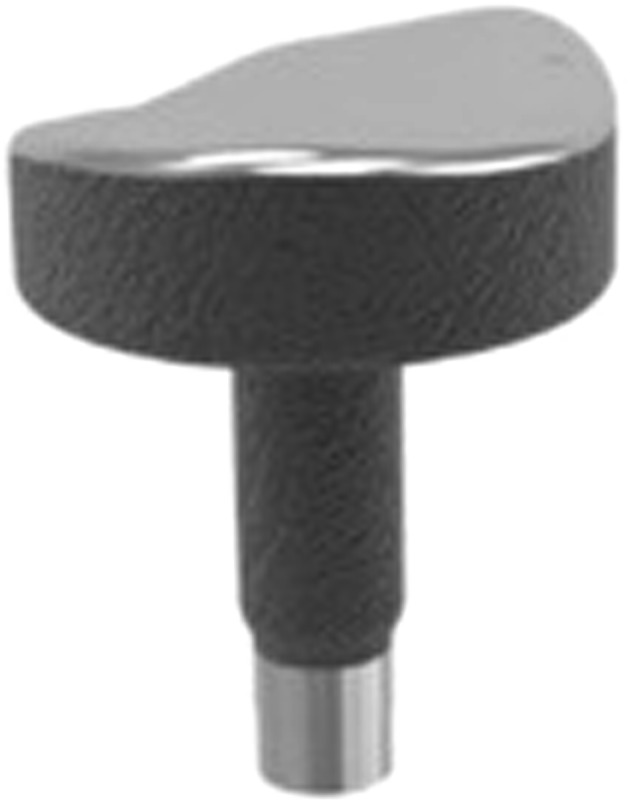
The Episealer Cr–Co implant with a customized circular shaped articulating surface and a hydroxyapatite coated peg for insertion

For this study a customized Cr–Co femoral condyle implant was manufactured by computer-aided design/computer-aided manufacturing (CAD/CAM) technique using MRI data. In addition, from the MRI data specific guide instruments were manufactured as part of the surgical kit to obtain accurate implant positioning in order to avoid damage to the opposing cartilage surface [20]. The Cr–Co implant has a 3- to 4-mm-thick circular shape where the articulating surface was contoured to precisely reconstruct each patient’s individual cartilage lesion and condylar contour. The implant has a 15-mm-long and 4-mm-thick peg that was inserted in an undersized drill hole to provide primary fixation. For secondary or long-term fixation a double coating of hydroxyapatite-on-top-of titanium was applied on the surfaces facing bone and cartilage (Fig. 1). This coating has been shown to give firm and consistent osseochondrointegration in an animal model [21].

### Pre-op planning

The patient-specific designs of the Episealer implants and surgical drill guides are based upon MRI imaging. The patient undergoes an MRI scan that is uploaded to a MRI processing system (μiFidelity^®^) through a web-based online management system. By processing MRI data, a 3-dimensional model of the damaged joint is recreated. The lesion is identified and the implant (Episealer^®^) and surgical drill guides (Epiguide^®^) are designed to enable the replacement of the damaged area with a fitting implant. The implant is available in four different diameters, 12, 15, 17 and 20 mm, depending on the condyle and lesion size.

### Operative technique and description of devices

The patients were administered cloxacillin 2 g 15–45 min before surgery and 2 h postoperatively. Surgery is performed under general or spinal anesthesia. The procedure started with a diagnostic arthroscopy to evaluate the joint and confirm absence of significant concomitant injuries and to validate the size of the injury. The femoral condyle is then exposed through a medial or lateral mini-arthrotomy. After exposure of the femoral condyle, an assessment of the cartilage injury was made ensuring that the implant was sufficiently large to cover the lesion. The drill guide was matched to the unique position on the femoral condyle surface with the lesion centerd in the drill guide. At least two surgical pins should be drilled at the rim of the femoral condyle to firmly fasten the drill guide on the condyle. The rims of the cartilage defect were adjusted with a cartilage cutter and the defect was drilled and milled in order to prepare a hole in the osteochondral tissue for implant insertion. Drill depth was adjusted to counter-sunk the implant 0.5 mm as indicated by a dummy (Epidummy). The depth was fine-tuned using an adjustment ring in the tool kit allowing incremental adjustment of 0.2 mm and the final implant position was controlled by a testing device (Epidummy). By inserting the implant, the defect was sealed by the metallic resurfacing implant hence reconstructing the original contour of the femoral condyle. The drillguide and pins were removed and the wound closed in layers. Postoperative pain alleviation by intraarticular ropivacaine was given. Anti-thrombotic medication was not routinely administered. Four different experienced knee surgeons at three different centers performed the surgery and practiced the technique on saw bones prior to the surgery.

### Rehabilitation

Week 1–2: crutches and non-weight-bearing, full passive and active exercises without resistance. Activation of quadriceps muscle in extension. Aims: full passive range of motion.

Week 3–6: crutches and partial weight-bearing. Cycling with light resistance. Straight leg lift. Initiation of core-stability training. Leg curls with light resistance. Aims: full active and passive range of motion. Full weight-bearing at week 6.

Week 7–12: full weight bearing, no crutches. Cycling with increasing resistance. Balance training. Squats and lunges with increasing resistance. Aims: normal walking. Good knee control. Managing ADL.

### Clinical and radiological evaluation

Primary endpoints assessments were frequencies of un-anticipated side effects such as surgical tools usability and functionality, ongoing osteoarthritis, implant migration, mechanical implant loosening, implant fracture, inflammation, no pain alleviation or allergic reaction. Secondary endpoints assessments were performance compared to base-line by patient reported outcome measurements (PROMS) preoperatively, at 3 and 6 months, 1 and 2 years; Knee injuries and Osteoarthritis Outcome Score (KOOS), EuroQoL (EQ-5D), Tegner Activity Scale (Tegner Score), In addition, Visual Analogue Scale (VAS pain) and knee range of motion (ROM) measurements were recorded.

### Radiostereometric analysis (RSA)

RSA is a high-precision method of assessing three-dimensional (3D) micro-movement from calibrated stereoradiographs and is a standard technique for evaluating new implants since early migration can predict loosening [9, 16, 24]. The RSA method in our study followed published guidelines for RSA [28] and the method developed for this particular implant [27]. Using this method the accuracy is between 0.08 and 0.19 mm and the precision 0.12–0.33 mm. During surgery 5–6 tantalum markers (1.0 mm) were placed in the bone surrounding the implant to serve as the reference segment for the RSA analysis. The tip of the implant, in the shape of a 3-mm hemi-sphere was used as the measured point of the prosthesis. Two days postoperatively, at 6 months, 1 and 2 years after surgery, the operated knee was then placed in a biplanar calibration cage (Cage 10; RSA Biomedical AB, Umeå, Sweden). Digital radiographs (Bucky Diagnostic; Philips, Eindhoven, the Netherlands) were then taken using one fixed and one mobile X-ray source. The exposure was set to 125 kV and 2.5 mAs. The radiographs were saved in a standard dicom file format (resolution 254 dpi) and uploaded to a workstation. UmRSA 6.0 computer software (RSA Biomedical) was used for all measurements and migration analyses. The markers in the distal femur form one segment and the micromotion of the tip of the implant was then evaluated. The 3D translations of the tip in relation to the femoral bone segment were calculated at each follow-up visit and compared with the immediate postoperative measurements. We also measured the maximum total point movement (MTPM), which is the 3D translation vector of the tip. At 1 year, we performed two examinations 15 min apart on all patients with complete repositioning of the X-ray tubes and the calibration cage. We calculated the precision as the 95% confidence interval (SD 1.96) of the difference between these examinations. For translation along the *x*-(transverse), *y*-(vertical) and *z*-[anteroposterior (AP)] axes, this was 0.20, 0.32 and 0.30 mm, respectively, and for the MTPM it was 0.33 mm, corresponding well with previous in vitro precision determinations [27]. For individual patients, any migrations above these threshold values means that a detectable migration has occurred of the implant in relation to the bone.

The study was approved by the local ethics committee Karolinska Institutet (2012/109-3171). Informed consent was obtained from all patients and the study was conducted in accordance with good clinical practice and the declaration of Helsinki.

### Statistical analysis

All data were analyzed using IBM SPSS Statistics for Mac, version 23 (IBM Corp, Armonk, NY, USA). Data for patient demographics and patient-related outcome measures are expressed as median and range. Group differences were analyzed with Mann–Whitney rank sum test, two-tailed. A *p* value of less than 0.05 was considered statistically significant.

## Results

### Patients

Thirteen of the screened patients fulfilled the inclusion criteria. Two patients chose not to proceed with surgery. On ethical grounds questions about reasons for drop-out could not be asked. For one patient the injury was per-operatively found to be more severe than anticipated, 24 mm, and could not be sufficiently replaced by the planned implant, 20 mm, and was therefore excluded. The remaining ten patients were followed for 2 years. Demographics, injury and surgical data as described in Table 1 with median age of 42.5 years (range 36–56), seven male and three female patients, seven ICRS grade 4 and three ICRS grade 3 injuries, implant size 17 mm in four patients and 20 mm in six patients. All surgeries were done on the medial femoral condyle. All patients had previous surgery in the involved knee. Seven patients had previous failed microfracture and three patients had previously had ACL reconstruction and minor medial meniscus injuries with small flap tears that were resected. If occupations with low physical demand, sick leave was short, but sick leave was necessary for up to 1 year with high demanding manual labor. Patient no. 6 was unemployed at time of surgery, but found work as a janitor 6 months after surgery.

**Table 1:** Demographics, injury and surgical data

Patients	Age (years)	Sex	BMI (kg/m^2^)	ICRS (1–4)	Involved knee	Implant size (mm)	Localization	Previous surgery	Surgery time (min)	Occupation	Postoperative sick leave (weeks)
1	56	Male	23	4	Left	20	Medial condyle	Microfracture	92	Sports teacher	7
2	42	Female	25	4	Right	17	Medial condyle	ACL rec. Minor medial meniscus resection	79	Office	6
3	36	Female	23	4	Left	20	Medial condyle	ACL rec. Minor medial meniscus resection. Microfracture	73	Office	6
4	39	Male	26	4	Right	20	Medial condyle	Microfracture	62	Blacksmith	36
5	42	Male	25	3	Right	20	Medial condyle	Shaving	53	Physiotherapist	12
6	49	Male	34	4	Right	20	Medial condyle	ACL rec. Minor medial meniscus resection. Microfracture	58	Unemployed	–
7	41	Male	27	4	Left	17	Medial condyle	Microfracture	94	Office and fireman	6
8	54	Male	30	4	Left	17	Medial condyle	Microfracture	67	Nurse	6
9	43	Female	30	3	Left	17	Medial condyle	Shaving	72	Hairdresser	100% 6 weeks 75% 20 weeks 50% 16 weeks 25% 4 weeks
10	44	Male	28	3	Right	20	Medial condyle	Microfracture	45	Chief executive officer	–

### Surgical usability of implant and instruments

Surgery time improved with increased experience and each subsequent procedure and reached below 60 min (Table 1). In two patients (nos. 1 and 2) the cartilage injury on the medial femoral condyle was localized lateral and close to the patella and trochlea which made it difficult to position the drillguide. The problem was solved by cutting the most anterolateral part of the drillguide which did not affect the stability of the guide and the continuing procedure.

### Radiographic evaluation

**Fig. 2:**
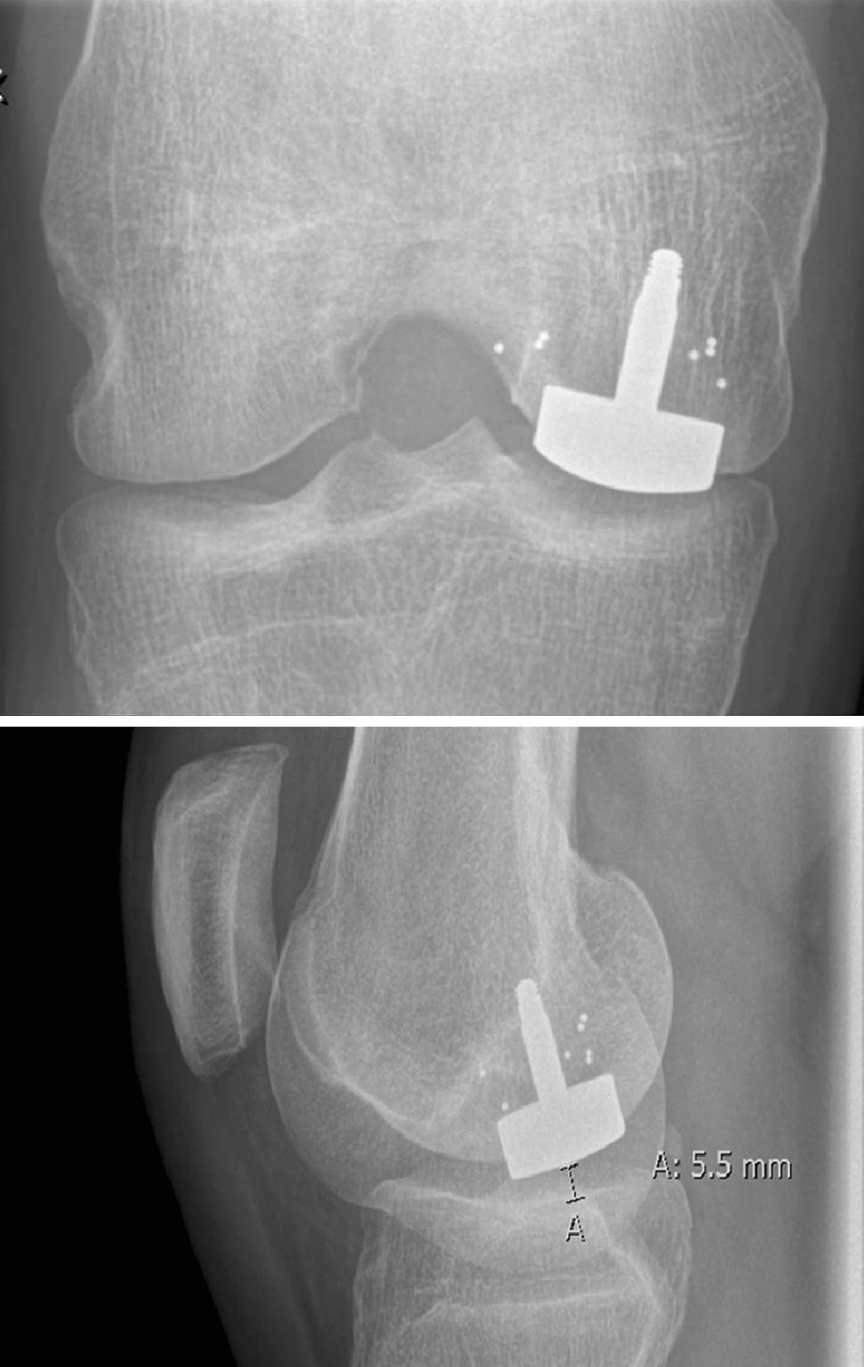
Radiographs, 2 years, patient no. 4

Radiographs at 2 years showed no peri-prosthetic radiolucency, cyst formation, implant subsidence or other signs of disassembly. No radiographic sign of damage to the opposing tibial cartilage or signs of osteoarthritis such as decreased joint space was observed (Fig. 2).

### Radiostereometric analysis

There were no missing RSA data on any of the follow-ups. Overall, we found very small migration and no implant migrated beyond the detection limit within 6 months postoperatively. After this period, one implant migrated between the 6 months and 1 year follow-up in the *z*-axes, but had stabilized at the 2-year follow-up. The mean overall migration was statistically significant compared to the postoperative value for MTPM but not for individual migrations in the *x*–*y* or *z*-axes (Table 2).

**Table 2:** Migration of the proximal tip of the implant measured with RSA compared to the 2-day postoperative value

Tip migration (mm)	Migration		*p* value
	Mean	SD	
Transverse (*x*)			
6 months	0.00	0.09	n.s
1 year	− 0.09	0.27	n.s
2 years	− 0.04	0.20	n.s
Vertical (*y*)			
6 months	− 0.03	0.12	n.s
1 year	0.03	0.26	n.s
2 years	0.00	0.20	n.s
Anteroposterior (*z*)			
6 months	0.03	0.10	n.s
1 year	− 0.12	0.36	n.s
2 years	− 0.06	0.15	n.s
MTPM			
6 months	0.16	0.09	< 0.001
1 year	0.32	0.43	0.04
2 years	0.27	0.16	< 0.001

### Patient-related outcome measures and range of motion measurements

**Fig. 3:**
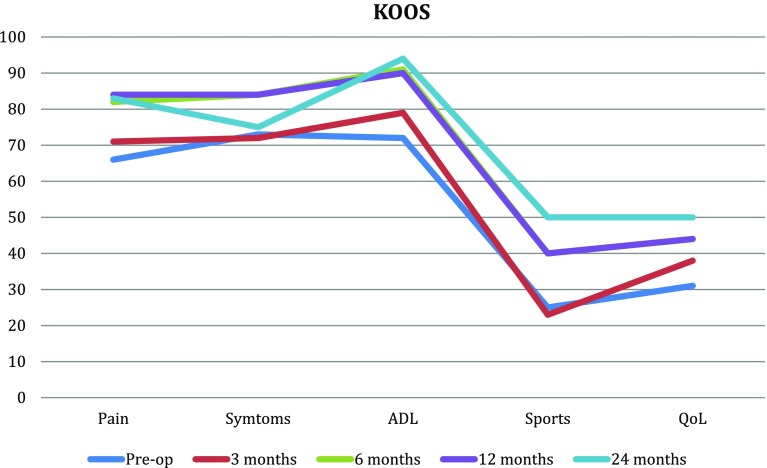
KOOS subscales at pre-op, 3, 6, 12 and 24 months

**Fig. 4:**
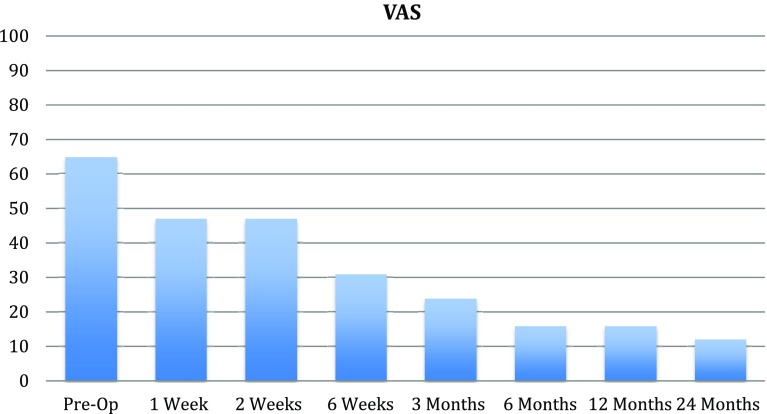
VAS at pre-op, 1, 2 and 6 weeks. 3, 6, 12 and 24 months

At 2 years all follow-up scores; VAS, EQ5D and KOOS showed improvements that reached significance for VAS (*p* ≤ 0.001), Tegner (*p* = 0.034), the KOOS subscores ADL (*p* = 0.0048), sport and recreation (*p* = 0.034) and quality of life (*p* = 0.037). VAS and KOOS scores improved gradually, with the greatest improvement between month 3 and 6 (Figs. 3, 4). The improvements in EQ5D did not reach statistical significance. No infections, deep venous thrombosis or other complications occured in the postoperative period. A second-look arthroscopy was performed in patient no. 2, 10 months after the index surgery due to persistent anterior knee pain. The arthroscopy showed slight patellofemoral osteoarthritis, ICRS 1–2. The implant was well fixed and cartilage laid over the edges of the implant. Range of motion reached preoperative values for all patients at 6 weeks post-surgery.

## Discussion

Good subjective outcome and no implant migration were shown in this first clinical report of a new customized focal knee resurfacing system, the Episealer^®^. The middle-aged patients, recently noted as the “gap”-patient [19], with a disabling focal cartilage injury in the knee is a challenging group. Common cartilage procedures have shown inferior results in this group of patients and a conventional arthroplasty poses great risk of need for revision surgery due to implant loosening and wear [11, 13–15, 23]. The patients reported here had had a number of previous procedures during long periods of time and were in a salvage situation. It was shown that the Episealer^®^ device was an effective treatment option for focal cartilage lesions in these highly symptomatic middle-aged patients. The short-term patient-related outcome measures showed good-to-excellent results and was in accordance with previously reported results from other focal knee resurfacing systems [7, 29]. Further follow-up to ensure continued good clinical results at long-term is however mandatory.

The purpose of the study was primarily to ensure good implant safety and surgical usability of the implant and instruments. In two patients the drill guide was hard to position since the cartilage defect was in close proximity to the patella and trochlea. Trimming the anterolateral part of the guide was necessary and that allowed good guide stability and positioning. The surgeries were performed by experienced knee surgeons, but it should be noted that there is a learning curve and surgery time decreased with each subsequent procedure. Surgery time was affected by the study protocol, check list procedures and thorough documentation necessary to ensure good patient safety with this new procedure.

In one patient MRI underestimated the size of the injury and the implant was to small to fit the damaged site. A reassessment of the MRI examination was made postoperatively and this prompted changes in the MRI evaluation procedures and no incidents with implants not matching the site of the injury has occured thereafter.

In one patient a second-look arthroscopy was performed after 10 months. The implant showed good osteochondral integration and no signs of implant-related injuries to the adjacent tibia plateau. The prosthesis appeared well incorporated.

To our knowledge, there are no previous publications on RSA and knee resurfacing implants. The migration of our implant is well below (non-detectable) the proposed limit of 0.3 mm, which is predictive of late failure of a total knee prosthesis [24]. Only one patient had migration up until 1 year, but was stable after 2 years. This indicates that the implant is stable enough under an extended period of time for osseo-integration to occur. The limitations of our RSA method when measuring only the tip of the implant as a proxy for overall migration, has been well described in the methods paper for this prosthesis [27]. To acquire more thorough migration measurements, including rotations, newly developed methods using three-dimensional computed tomography RSA would be required for these types of implants [4]. At the initiation of the current study, this was not available but can possibly be used in future studies where small implants like these are used and where RSA marking is not possible.

Radiographic exams at 12 months showed no signs of tibial damage or progressing osteoarthritis. It is recognized that focal knee resurfacing with metal implants warrants correct positioning in order to avoid injuries to the opposing tibia and subsequent cartilage degeneration [2, 20]. Previous mid-term follow-up of focal knee resurfacing with metal implants have shown risk of osteoarthritis progression with more than 20% revision to knee arthroplasty within 7 years [17]. A similar focal knee resurfacing method with a metal implant has shown progressing radiographic Kellgren–Lawrence osteoarthritis scores even at short-term follow-up [8]. However, it has been stipulated that correct implant placement does not lead to increased contact pressures [1, 3]. The purpose with the development of an individualized implant and instrumentation system was to achieve accurate and consistent implant position at a correct angle and depth, thereby minimizing the risk for a misaligned and protruding implants in order to avoid further cartilage damage with the expectation to maintain the good results even at long-term.

## Conclusion

This new customized focal knee resurfacing implant shows good implant safety and patient satisfaction, function as well as pain is significantly improved. RSA did not detect any implant migration. A limitation of this study is that it is a short-term follow-up and it must be emphasized that the small group of patients do not allow generalization of the results and further follow-up and research is mandatory. The implant should be used cautiously by experienced surgeons.
